# Reversible fluorescent solid porous films for detection of zinc ions in biological media

**DOI:** 10.1186/s13036-025-00484-1

**Published:** 2025-02-18

**Authors:** Alessandro Gandin, Laura Brigo, Sujatha Giacomazzo, Veronica Torresan, Giovanna Brusatin, Alfredo Franco

**Affiliations:** 1 Department of Industrial Engineering, University of Padova, Padova, 35131 Italy; 2 INSTM Padova RU, University of Padova, Padova, 35131 Italy; 3 Department of Applied Physics, University of Cantabria, Santander, 39005 Spain

**Keywords:** Zinc ion, Zinpyr-1, Fluorescence sensors, Hybrid films, Reversible sensors

## Abstract

The need for a sensitive, selective, non-invasive and reversible fluorescent sensor for Zn^2+^ monitoring is addressed in this work. A novel guest-host system is developed, including a Zn^2+^ sensitive fluorescent probe, Zinpyr-1, embedded in a porous optically transparent hybrid film. The entrapped probe molecules are accessible and can interact with the external analyte. The immobilized Zinpyr-1 confirms its specificity and selectivity for Zn^2+^, as shown by sensing tests conducted in buffer solutions that mimic the ionic composition of biological media. The uniqueness of the developed sensor system lies in its reversibility, combined with a fast and selective response, allowing dynamic measurements of zinc concentrations in the 1 µM to 1 mM range within few tens of seconds. Unlike most Zn^2+^ sensors, this system is a film-based sensor, making it an interesting minimally invasive tool for future studies on how live cells cultured on it dynamically regulate the Zn^2+^ concentration under controlled physiological conditions.

## Introduction

Zn^2+^ is the second most abundant transition metal in the human body, where, in free form, is always present as a divalent ion. Intracellular Zn^2+^ stores have been discovered in multiple tissues. Moreover, in several cell types (such as hippocampal and olfactory neurons, oocytes, and pancreatic β cells) secretory granules enriched in Zn^2+^ are present and undergo exocytosis, releasing Zn^2+^ extracellularly, in response to precise stimuli. The extracellular local concentration of Zn^2+^ after these secretory events is estimated to reach peaks as high as 300 µM. From intracellular to whole body level, Zn^2+^ homeostasis is dynamically and precisely regulated, and its impairment is associated with different pathological conditions [1]. Therefore, tremendous efforts have been made to develop sensitive analytical methods able to detect this cation with high temporal resolution [2]. 

Due to its 3d^10^4s^0^ electronic configuration, spectroscopic or magnetic signals do not occur for Zn^2+^, contrary to what is observed for other biological transition metal ions such as Fe^2+^, Mn^2+^, Cu^2+^. Therefore, common analytical techniques such as UV-visible spectroscopy, nuclear magnetic resonance (NMR) and electron paramagnetic resonance (EPR) spectroscopy cannot be applied, and fluorescence spectroscopy stands out as the method of choice [3, 4]. 

Starting from the 1980’s, a number of synthetic small-molecule fluorescent probes have been developed as selective for Zn^2+^ over other biologically abundant divalent cations [5] like Mg^2+^ [6], Fe^2+^, Mn^2+^ and Cu^2+^ [7]. They have different dissociation constants tailored to the system under investigation and are compatible with biological systems thanks to, among other properties, their water solubility [2]. A comparative study has shown that the Zinpyr-1 (ZP1) molecule, previously characterized by Burdette and co-workers [8], produces the highest fluorescence response to free Zn^2+^, compared to other commercially available dyes (FluoZin-3 AM, Newport Green DCF, and Zinquin ethyl ester) which are especially responsive to bounded zinc [9]. 

**Fig. 1:**
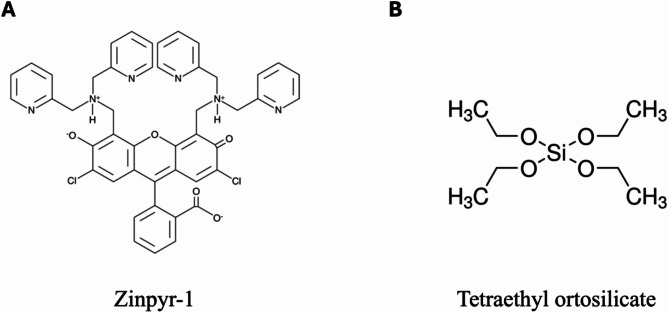
Chemical structures of: (**A**) Zinpyr-1 (ZP1), the used fluorescent probe for Zn^2+^ detection; (**B**) Tetraethyl ortosilicate, the precursor used for the synthesis of the films doped with ZP1 fluorophores via the sol-gel process

ZP1, whose chemical structure is shown in Fig. 1A, is a cell membrane-permeable fluorophore containing dipicolylamine as Zn^2+^ chelator. It has a K_d_ of 0.7 ± 0.1 nM for Zn^2+^, with a Hill coefficient close to 1, indicating a 1:1 probe-ion binding ratio. At physiological ionic strength and pH, its maximum excitation wavelength is 515 nm in absence of Zn^2+^, shifted to 507 nm if saturated with zinc ions. ZP1 molecule undergoes protonation of the electron donor amino groups in solution and a consequent deprotonation of the same group with Zn^2+^ binding [10]. 

An important requirement for the study of Zn^2+^ secretion in biological systems is the ability to perform dynamic measurements in live samples. Thus, the sensor should not interfere with biological processes, should be located as close as possible to the Zn^2+^-secreting cells and should provide a fast and reversible response to changing ion concentrations. Previous solutions for these applications opted for the use of molecular probes dissolved in culture medium (like the cell-impermeant FluoZin-3) [11] or anchored to the cellular membrane (like ZIMIR) [11] or genetically encoded (like GZnP) [12]. However, all these methods could interfere with the biological response.

The alternative strategy we are reporting here is based on the immobilization of fluorescent probes into porous sensor films. Considering a culture area, these functional films are suitable for being deposited in the immediate vicinity of cells, minimizing fluorophore interference with biological processes, increasing the ease of handling, reducing the amount of required fluorophore, and protecting probes from potential interfering compounds. Moreover, a solid sensor film integration within a lab-on-chip culture system would allow extremely accurate dynamic measurements [13, 14].

Adsorption of the biomolecules to solid supports and entrapment within a polymeric network are common immobilization techniques, but they suffer many disadvantages such as leaching and loss of activity and stability with time [15, 16].

The sol-gel process offers mild synthesis conditions for the direct preparation of functional hybrid systems, thanks to the possibility of tuning sol composition according to specific requirements. Starting from a colloidal suspension, the sol-gel material is obtained by hydrolysis and polycondensation of precursor molecules, a process that occurs at low temperature [17]. Owing to the porous nature of the material obtained through the sol-gel process, fluorescent probes can be entrapped during the production of the material, or can be covalently bound to the hybrid material network, and remain accessible to the external analyte [15]. The final material can be deposited in the form of films with good mechanical and optical properties, chemical stability, and high specific surface area thanks to a controlled porosity.

Here, fluorescent sensor films for Zn^2+^ detection suitable for the measurement of rapid concentration dynamics were developed. ZP1 fluorescent probes were included into porous sol-gel films, made of hybrid networks synthesized starting from tetraethyl ortosilicate (TEOS) as SiO_2_ precursor **(**Fig. 1B**)** [18].

When exposed to Zn^2+^ ions in solution, the system showed selective response down to nanomolar concentrations, with response times in the order of tens of seconds. Distinctively, the sensor films demonstrated full reversibility, without the use of chelating agents, which, to the best of our knowledge, is a crucial turning point in the field of Zn^2+^ sensors.

## Materials and methods

### Synthesis and film preparation

The system was synthesized through sol-gel chemistry starting from tetraethylortosilicate (TEOS) as SiO_2_ precursor. A solution of TEOS (Sigma-Aldrich, reagent grade, 98%), EtOH (Sigma-Aldrich, ACS reagent, 99.5%) and bi-distilled water was mixed in a monomer: H_2_O = 2:3 molar ratio, using HCl 1 M (Sigma-Aldrich, ACS reagent, 37%) as catalyst. Long-range ordered mesopores were produced within the films through self-assembly of the ionic surfactant CTAB (Sigma-Aldrich, 98%), which is added to the solution at critical micelle concentration (4–5 wt%) [19]. Specifically, a pre-hydrolyzed stock solution was first made mixing TEOS, EtOH, bi-distilled water and HCl at the following molar ratios: TEOS: EtOH: H_2_O: HCl = 2:8:3:1 × 10^− 4^, and keeping the mixture for 90 min under reflux at 60 °C. Secondly, a sol is formed stirring together 1 ml of pre-hydrolyzed stock solution, 136 µl of HCl and 46 µl of bi-distilled water for 15 min and ageing at room temperature for additional 15 min. The third step is a dilution of the sol, stirring together 1.182 ml of the sol and 2.6 ml of EtOH for 15 min and ageing the solution for 48 h at room temperature. Finally, 4.5 wt% of CTAB was added to the diluted sol and stirred for 15 min at room temperature. The films were deposited by spin coating on fused silica glass slides, with final thickness of 200–400 nm. The glass slides were previously washed with piranha solution (H_2_SO_4_ and H_2_O_2_ in 3 to 1 volume ratio) and the spin coating was carried out at 2000 rpm for 20 s using an SCS Pi-Kem G3P spin-coater.

### Nanostructure fabrication and surface functionalization

Sinusoidal nanometric and micrometric structures were obtained in the films through the soft-lithography patterning method. PDMS molds were fabricated using the Sylgard 184 Silicone Elastomer Kit (Dow Corning), which consists of a curing agent and a pre-polymer that have to be mixed in 1 to 10 weight ratio. The mixture was then outgassed in a desiccator connected to a vacuum pump for one hour, subsequently poured on nano-/micro-structured masters, and thermally cured at 70 °C in oven for about 1 h to promote polymerization before peeling the elastomeric replica off the masters. PDMS replicas were gently pressed with a finger on fresh-deposited films, and the assembly was cured with a 30 min thermal treatment at 80 °C in an oven, before delicately peeling the mold off the sample. Films were left overnight at 80 °C to complete condensation, then the templating agents were removed from films in oxygen atmosphere by washing the samples at 50 °C for 12 h in a solution made of EtOH (5 ml) and HCl (3 ml). Then, the films were functionalized with ZP1. First the films were immersed, for 20 h at 70 °C, in a 0.2 mM solution of aminopropyltriethoxysilane (APTES) (Sigma-Aldrich, 97%) and toluene (Sigma-Aldrich, anhydrous, 99.8%). Then the films were washed with toluene and ethanol and dried for 1 h at 70 °C. Separately, 160 µl of ZP1 (Santa Cruz Biotechnology, 95%) diluted in chloroform at 2.4 mM was mixed with a solution of 100 mM:100 mM ratio of N-3-(dimethylaminopropyl)-N’-ethylcarbodiimide (EDC) (Sigma-Aldrich, 97%) and N-hydroxysuccinimide (NHS) (Sigma-Aldrich, 97%) in buffer at pH 7.5. The buffer composition is: 1 L of de-ionized water, 2.603 g of HEPES (Sigma-Aldrich, 99.5%), 6.954 g of NaCl (Sigma-Aldrich, ACS reagent, 99%), 0.35 g of KCl (Sigma-Aldrich, ACS reagent, 99%), 0.105 g of MgCl_2_·6H_2_O (Sigma-Aldrich, ACS reagent, 99%), 0.163 g of KH_2_PO_4_ (Sigma-Aldrich, ACS reagent, 99%), 2.603 g of NaHCO_3_ (Sigma-Aldrich, ACS reagent, 99.7%), 0.547 g of CaCl_2_·2H_2_O (Sigma-Aldrich, ACS reagent, 99%). Finally, the films were immersed for 30 min in the ZP1 solution and washed with Milli-Q water.

### Material characterization

The obtained hybrid silica films were characterized by small angle x-ray diffraction, using a Philips PW 1729 diffractometer equipped with glancing-incidence X-ray optics. The analysis was performed at 0.5° incidence, using Cu Kα-Ni filtered radiation at 30 kV and 40 mA. The average d-spacing for the hexagonal lattice made of mesopores in the silica films was calculated using the Bragg equation. Given the transparency of the films within the investigated spectral range, their refractive index and thickness were determined using a J. A. Woollam Co. VASE spectroscopic ellipsometer, with the data fitted to the Cauchy dispersion relation. Optical absorbance spectra were recorded by Jasco V-570 and Jasco V-650 spectrometers in the range of 200–1500 nm or 200–900 nm, respectively. A morphological characterization of the nanostructured surfaces was performed by Atomic Force Microscopy (AFM, NT MDT Spectrum Instruments - model Solver Pro), acquiring images of sample areas of 3 × 3 and 10 × 10 µm^2^. The peak-to-valley height of the profiles was estimated from the Abbot curve as the height variation between the values corresponding to the 3% and 97% of the bearing area. The period was calculated from the position of the peak in the Fourier spectrum of the grating profile, obtained by averaging over scanned lines (orthogonal to the grooves) in AFM images.

### Sensing measurements

Zn^2+^ sensing was performed by fluorescence spectroscopy both in water and in buffer solution. The pH of the HEPES based buffer was 7.5. Zinc acetate was used as Zn ion source. Emission spectra were recorded by a Jasco FP-6300 spectrofluorometer with excitation and emission bandwidth of 5 nm, data pitch of 1 nm, and scanning speed of 200 nm/min. In order to reduce the contribution of the reflected radiation from collected spectra, films were placed in a cuvette filled with medium, forming an angle of incidence of 60° (maximum reflection would be detected at 45°).

## Results

### ZP1-doped sol-gel film characterization

Using sol-gel process and self-assembly strategies, a mesoporous system was synthesized.

**Fig. 2:**
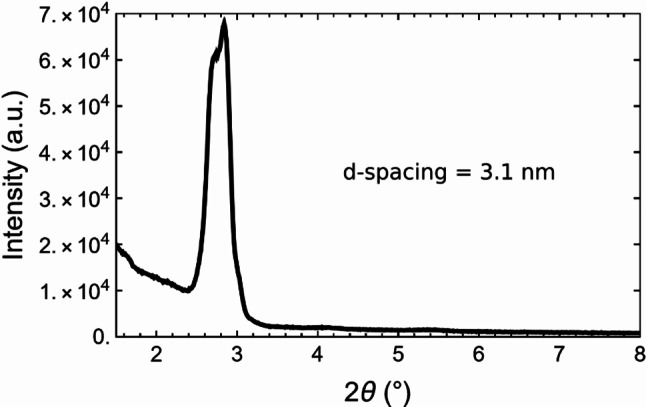
Representative small-angle XRD pattern of films synthesized from TEOS using CTAB surfactant as template, after CTAB removal. The XRD pattern is typical of a long-range ordered hexagonal distribution of pores, with a maximum at 2θ = 2.8 corresponding to a d-spacing of 3.1 nm. The XRD pattern was measured using a Philips PW 1729 diffractometer equipped with glancing-incidence X-ray optics, the incidence angle was 0.5°, and Cu Kα-Ni filtered radiation was used at 30 kV and 40 mA. The synthesis is well-controlled ensuring the same XRD pattern in 10 different films

The system was synthesized starting from TEOS as precursor and using cetyltrimethylammonium bromide (CTAB) as template. Long range ordered structures were obtained through the combination of bottom-up and top-down approaches. The surfactant self-assembly was exploited to induce the formation of periodically ordered mesopores within the SiO_2_ network [20], while soft lithography was used to induce the formation of a surface sinusoidal pattern [21], to improve the accessibility to the mesopores. Figure 2 shows small angle X-ray diffraction spectra of TEOS films after template removal. Considering the Bragg peak at 2θ = 2.8, the material is characterized by a hexagonally ordered pore structure with average pore d-spacing of 3.1 nm.

**Fig. 3:**
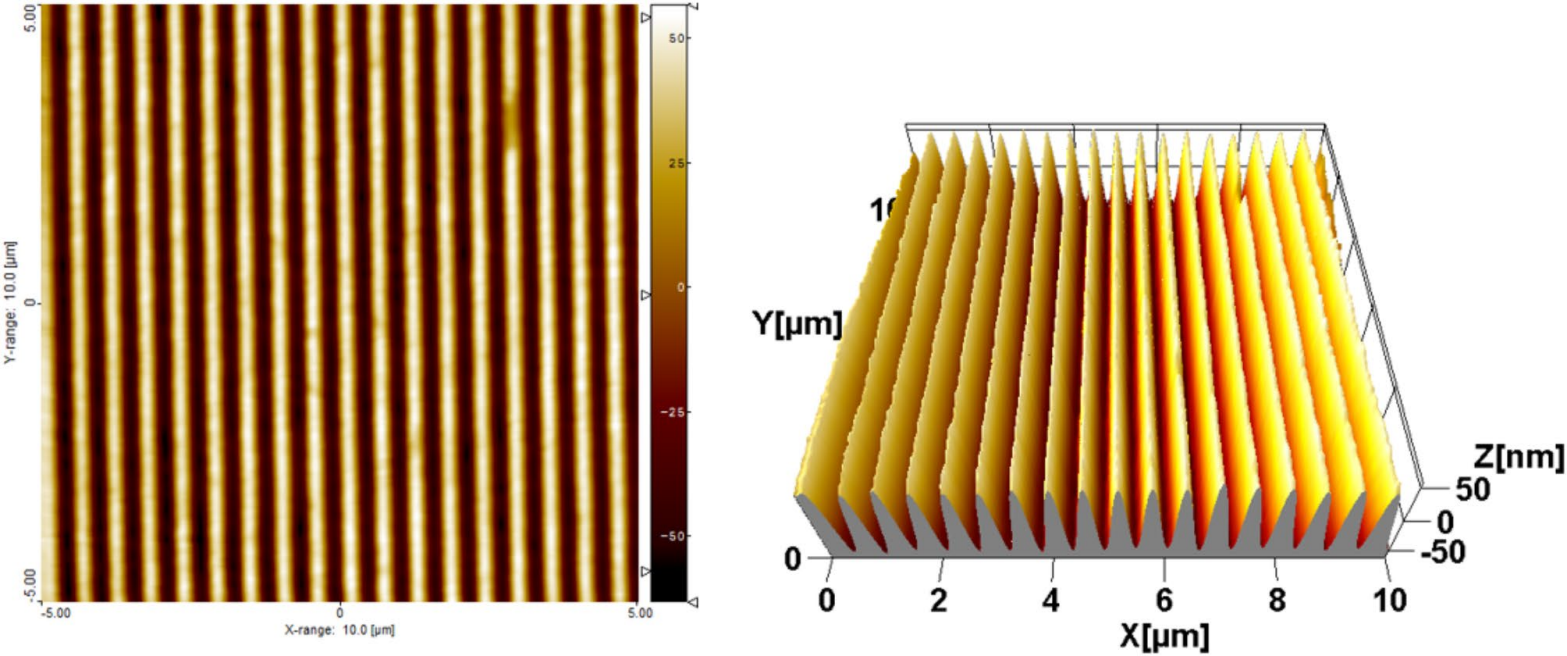
2D and 3D representation of AFM images obtained for the sinusoidal patterns created on the top of the mesoporous films, in order to increase their surface area and thus the fluorophore accessibility. The left-hand side shows a false-color 2D map to make evident that the period of the pattern is 560 ± 3 nm. The right-hand side shows a 3D representation of the film to show that the peak to valley height is 120 ± 6 nm. These values are the average of 10 different samples. The measurements were performed through Atomic Force Microscopy (AFM, NT MDT Spectrum Instruments – model Solver Pro), acquiring images of sample areas of 3 × 3 and 10 × 10 µm^2^. The peak-to-valley height of the profiles was estimated from the Abbot curve as the height variation between the values corresponding to the 3% and 97% of the bearing area. The period was calculated from the position of the peak in the Fourier spectrum of the grating profile, obtained by averaging over scanned lines orthogonal to the grooves

Figure 3 reports AFM images of a surface sinusoidal pattern transferred into the mesoporous film: the peak to valley height is of 120 ± 6 nm and the period is of 560 ± 3 nm, as estimated after measuring multiple samples.

**Fig. 4:**
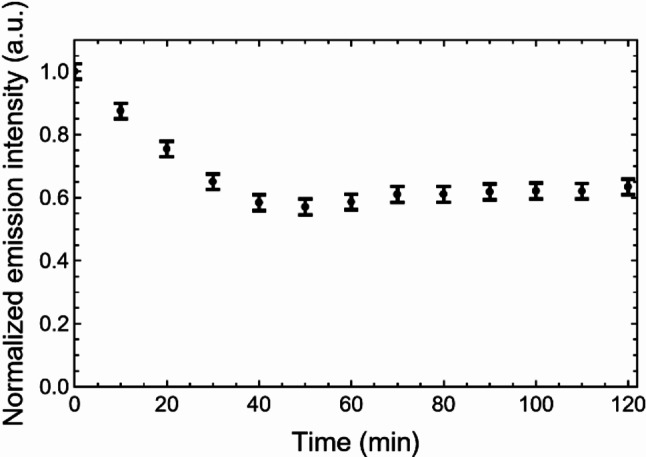
Leaching test performed on nanostructured sinusoidal films made of mesoporous hybrid SiO_2_ doped with ZP1 fluorophores. The error bars correspond to the maximum uncertainty from 5 independent tests

Leaching tests were performed through fluorescence spectroscopy to assess the effective film functionalization with selective probes in water environment. They showed that the fluorescence emission was stable after immersion of the film in liquid for more than one hour, after a fluorescence reduction in the first 30 min due to the removal of dye adsorbed to the film surface, and not entrapped within the silica network or covalently bound to it **(**Fig. 4**).**

### Optical characterization

The optimal fluorophore concentration was investigated to maximize the fluorescence signal resulting from ZP1 probes in the hybrid films, considering possible effects of fluorescence quenching and limits ascribed to the used optical system.

**Fig. 5:**
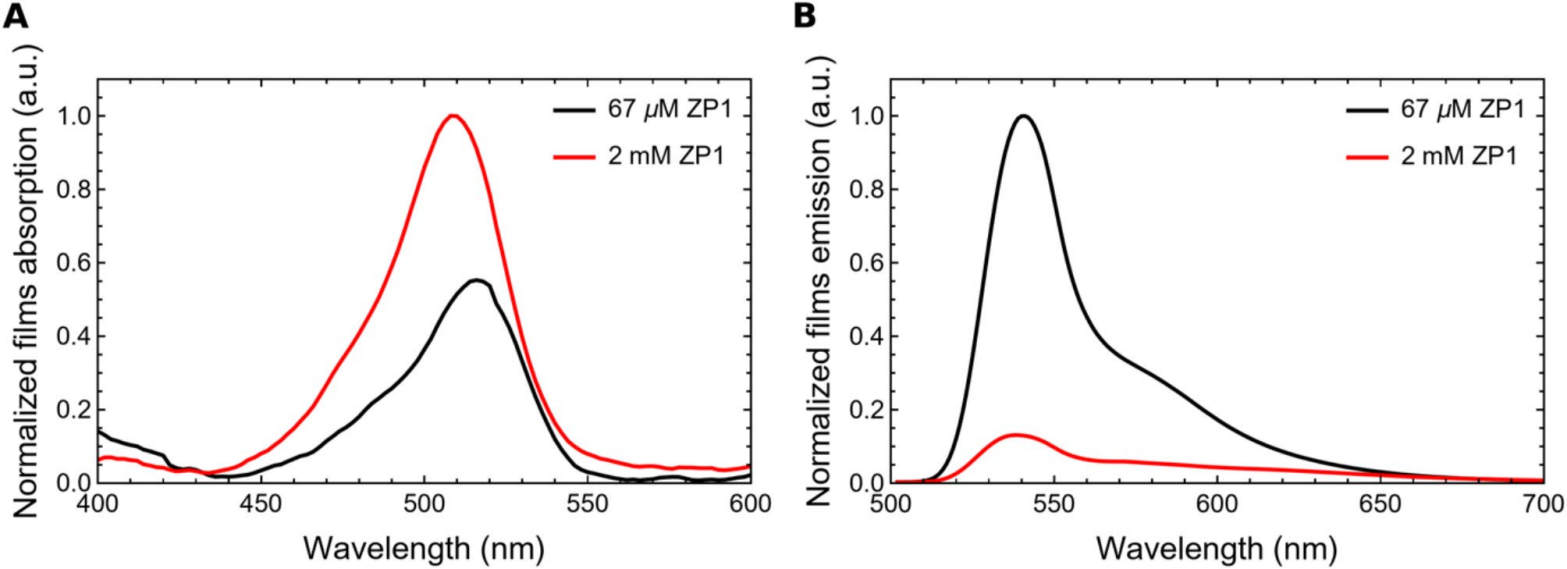
Normalized absorption (**A**) and emission (**B**) spectra, excited at 492 nm, of mesoporous silica films prepared with the same thickness (350 nm) but with two different ZP1 concentrations: 2 mM and 67 µM. The highest fluorescence emission signal is observed in the films prepared with lower fluorophore concentration, in absence of Zn^2+^ ions

Figure 5 shows normalized absorption and emission spectra of mesoporous silica films embedding ZP1 molecules. As the zinc ion detection mechanism is based on monitoring variations in fluorescence emission intensity, the best conditions to maximize signal-to-noise ratio and thus sensor sensitivity were obtained for a concentration of 67 µM, among tested ZP1 concentrations in silica films.

### Dynamic sensing tests

**Fig. 6:**
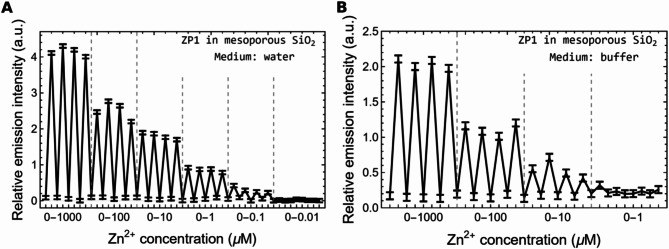
Zn^2+^ sensing performed with mesoporous nanostructured silica films in water (**A**) and in buffer (**B**) solutions presenting variable ion concentrations, after regeneration steps in the respective medium (the regeneration step is performed flushing the film with milli-Q water for 5 s). The segments connecting experimental points in the scatter plots have no physical meaning and are reported only to ease the visualization of sensing dynamics. For λ_exc_ = 492 nm, the peak position is at λ_em_ = 536 nm. Error bars are determined by the scale resolution of the sensor

Zn^2+^ sensing measurements were performed, both in water and in buffer solution, for films with a ZP1 concentration of 67 µM. These experiments aimed at two purposes: first, confirming ZP1 maintains its well-known sensitivity and selectivity against other ions once embedded within the fluorescent sensor films; and second, demonstrating the reversibility of Zn^2+^ detection of the sensor film without the use of chelating agents. The obtained results for the nanostructured mesoporous SiO_2_ films are reported in Fig. 6.

The plots show a variation in intensity of the fluorescence emission peak, after consecutive immersions of the film in the reference medium (Zn^2+^ free medium) and in the same medium containing different concentrations of Zn^2+^ ions, from 1 nM to 10 mM. The reported relative emission intensity is calculated as: (Iem–Iem-medium) / Iem-medium, where Iem is for the intensity of the fluorescence emission peak in the medium containing a given concentration of Zn^2+^ ions, while Iem-medium is for the intensity of the fluorescence emission peak in the reference medium. The reversible behavior arises due to the covalent functionalization of the ZP1 molecules to the solid substrate through their benzene moiety. This approach has two major effects that drastically change the behavior of the ZP1 molecules if compared with a homogeneously dispersion within the reference medium: it decreases their pKa value [22] and keeps the ZP1 fixed under the flow of the reference medium which can remove the Zn^2+^ ions from the ZP1 molecules thus regenerating the sensor.

Concentrations of Zn^2+^ in water down to 0.1 µM can be detected by the mesoporous silica films with periodic nanostructured surface, while in buffer solution, the lowest limit of detection is 10 µM. The investigated dynamic range is 0.01 µM to 1 mM.

The system can detect zinc ions with response times in the order of tens of seconds. In fact, spectra are measured right after immersing films in the medium, and the time required to collect them is of few tens of seconds. Distinctively, the sensors operate in a reversible way, without necessity of introducing chelating agents, and regeneration times are comparable to response times for the used readout system. The experimental setup does not allow dynamic measurements along the first tens of seconds, but the emission signal remains stable after such time. Further studies are needed to determine the lowest interval at which the sensor detects changes in Zn^2+^ concentration. However, to assess the practical applicability of the sensor, a two month-long stability test was conducted. The sensor was kept in vacuum desiccator under darkness for two months, after which its response remained stable. Furthermore, as a proof of concept, the sensor’s reversibility was successfully demonstrated in a simple, home-made microfluidic device featuring a single straight channel through which the aqueous sample flowed continuously across the sensor surface. The channel, made of polydimethylsiloxane (PDMS), had dimensions of 200 μm in height, 1 mm in width, and 26 mm in length, with a flow rate of 0.05 mL/s controlled by a syringe pump (New Era NE-300 InfusionONE). The details of this test are not included in the present work.

The sensor is selective against Na^+^, K^+^, Mg^2+^, Ca^2+^ cations, up to concentrations of 151 mM, 6.2 mM, 0.5 mM, 4 mM, respectively, as the data from the measured basal fluorescence in buffer indicates. Besides, it is reported that the ZP1 is highly selective against Mn^2+^, Fe^2+^, Co^2+^, Ni^2+^ and Cu^2+^ divalent cations as well as against various concentrations of phosphate derivatives [23–25].

**Fig. 7:**
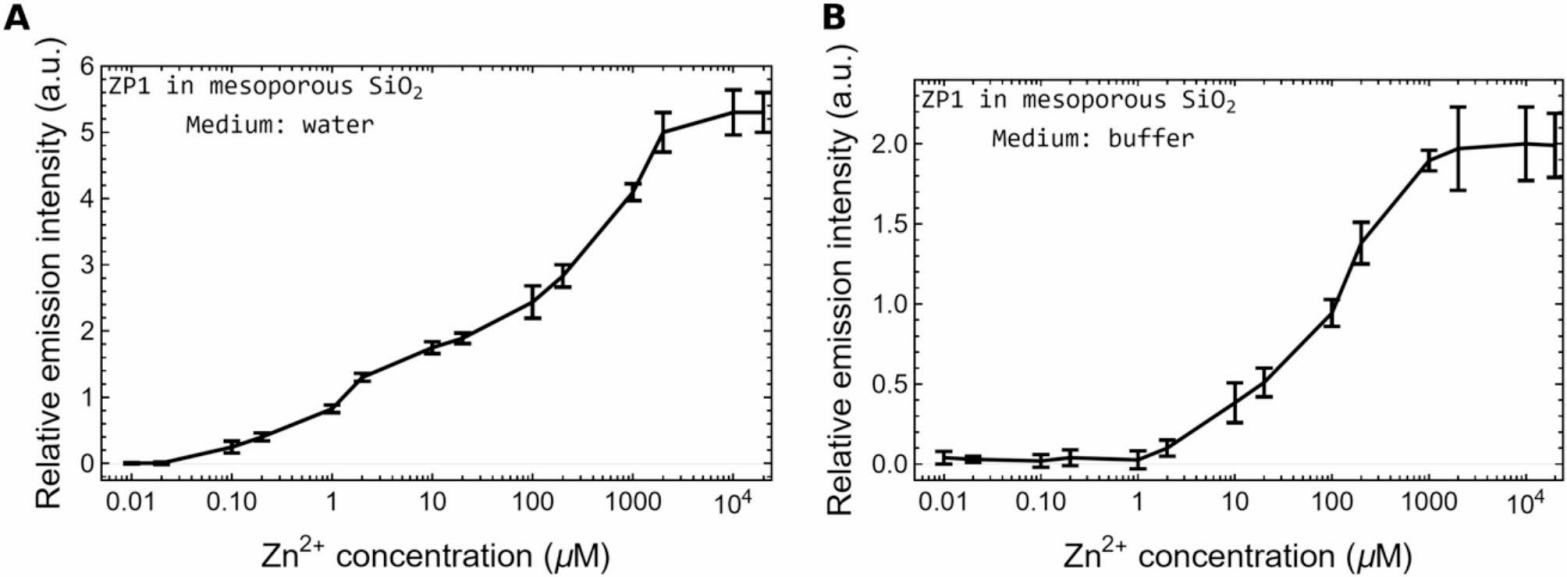
Response trend plots for the sensor under water (**A**) and buffer (**B**). The plots cover a broad range of Zn^2+^ concentrations from the lowest limit of detection to the saturation concentration. The sensor sensitivity was determined through the sensor linear response, in such a way the sensitivity for Zn^2+^ in water is 9.83% of its maximum emission intensity per µM and 0.25% of its maximum emission intensity per µM, for Zn^2+^ in buffer. The lowest limit of detection was determined from the lowest measured intensity that is clearly different to the basal fluorescence intensity: it was 0.1 µM for Zn^2+^ in water and 10 µM for Zn^2+^ in buffer. The error bars represent the standard deviation of the data reported in Fig. 6

The sensor response trends under water and buffer can be seen in Fig. 7. The sensor sensitivity was determined through its linear response as 9.83% of its maximum emission intensity per µM, for Zn^2+^ in water, and 0.25% of its maximum emission intensity per µM, for Zn^2+^ in buffer. The lowest limit of detection was determined from the lowest measured intensity that is clearly different to the basal fluorescence intensity: it was 0.1 µM for Zn^2+^ in water and 10 µM for Zn^2+^ in buffer. As reported in Fig. 7, the emission intensity in buffer is lower than in water. This is an expected behavior for this molecule since its response depends on the pH of the medium, with a typical quantum yield of 0.38 in water at pH 7 which decreases as the pH values increase, as is the case of our buffer at pH 7.5 [26].

## Discussion

While a large number of molecular sensors for Zn^2+^ ions in aqueous or polar organic solvents have been described in the literature, very few examples of sensor films for Zn^2+^ detection are reported. Kim D. W. et al. [27] developed selective film sensors made of self-assembled monolayers of receptors on quartz plat surfaces, but they were not natively reversible, showed a longer response time (110 s) and were not suitable to be used in aqueous media. Ying Z. et al. [28] used a spiropyran derivative incorporated in PVC films for highly sensitive (detection limit of 1.5 × 10^− 7^ M) and selective fluorescent sensing of Zn^2+^ ions in a wide dynamic range (from 5 × 10^− 7^ to 4 × 10^− 4^ M), in this case the sensor stability was a solved critical issue, but once more the system was regenerated only in the presence of chelating agents (ethylenediaminetetraacetic acid, EDTA). Shellaiah M. et al. [29] reported pyren-based organic thin-film transistors for real-time Zn^2+^ detection; however, their preparation is complex, and the detection process is not entirely optical-based. Surface plasmon resonance film sensors and optical fiber sensors were also studied as alternative to fluorescent films for a fast and sensitive Zn^2+^ ion detection (response times of 30–90 s, and detection limits < 0.1 ppm, ~ 1 µM) [30–32] but a sensor regeneration step was always necessary to record dynamic variations in analyte concentration.

For rapid and user-friendly Zn^2+^ ion detection, paper-based fluorescence sensors made of polyethyleneimine-derived fluorescent conjugates were reported too, however they require EDTA for regeneration [33].

Thus, considering the Zn^2+^ sensors reported in the literature (both molecular and film sensors) and the functional films developed in this work, the last ones (ZP1-doped nanostructured mesoporous silica films) exclusively demonstrated the ability to detect Zn^2+^ in a reversible manner with fast kinetics, and their concentration range is also of relevance in a physiological environment.

Table 1 compares the lowest limit of detection (LOD) of each of the discussed solid film sensors for Zn^2+^ detection. It is noteworthy that the performance of the sensor reported in this work is comparable with the performance of other sensors, with the advantage of a reversible response without the use of a chelating agent. For reference, the lowest limit of detection of Zn^2+^ ions of a recently reported fluorescent probe in aqueous solutions is 229 nM [34]. Under the same experimental conditions described in this study, the lowest limit of detection for ZP1 in water is 50 nM (with a 1 µM aqueous solution of ZP1, excitation wavelength of 492 nm, and emission wavelength of 525 nm).

**Table 1:** Lowest limit of detection for each one of the discussed solid film sensors for Zn^2+^ detection

Reference	[27]	[28]	[29]	[30]	[31]	[32]	This work	This work
LOD	170 µM	0.15 µM	5.46 µM	1 µM	0.7 µM	0.15 µM	10 µM	0.1 µM
Medium	Buffer	Buffer	Water	Water	Buffer	Water	Buffer	Water

## Conclusions

Engineered porous silica-based materials were produced by a sol-gel route and doped with ZP1 fluorophore to enable zinc ion selective detection. Nanostructured mesoporous hybrid films were investigated. The system can detect Zn^2+^ ions with response times in the order of tens of seconds. Distinctively, the exhibited dynamic response in water and in buffer solution demonstrates that the sensor is reversible. Nanostructured mesoporous silica-based films exhibit a lowest limit of detection of 100 nM, which is within the biologically relevant concentration range. These results pave the way for integrating sensor films into microfluidic devices, under dynamic flow of biological fluids. This represents a significant advantage over conventional sensors, which typically measure extracellular Zn^2+^ using fluorescent probes in solution, thereby limiting their use in microfluidic devices. Envisioned applications include, for example, the study of Zn^2+^ secretion from pancreatic β-cells, which is dynamically co-released with insulin, or the investigation of hippocampal and olfactory neurons activity.

## Data Availability

No datasets were generated or analysed during the current study.

## Abbreviations

ZP1: Zinpyr-1
TEOS: Tetraethylortosilicate
CTAB: Cetyltrimethylammonium bromide
EtOH: Ethanol
PDMS: Polydimethylsiloxane
APTES: Aminopropyltriethoxysilane
EDC: N-3-(dimethylaminopropyl)-N’-ethylcarbodiimide
NHS: N-hydroxysuccinimide
HEPES: 4-(2-hydroxyethyl)-1-piperazineethanesulfonic acid
AFM: Atomic force microscopy
